# Review article: Use of ultrasound in the developing world

**DOI:** 10.1186/1865-1380-4-72

**Published:** 2011-12-07

**Authors:** Stephanie Sippel, Krithika Muruganandan, Adam Levine, Sachita Shah

**Affiliations:** 1 Department of Emergency Medicine, Brown University, 593 Eddy Street, Providence RI, 02903, USA; 2 Department of Emergency Medicine, University of Washington Medical Center,1959 NE Pacific Street, Seattle, Washington, USA

## Abstract

As portability and durability improve, bedside, clinician-performed ultrasound is seeing increasing use in rural, underdeveloped parts of the world. Physicians, nurses and medical officers have demonstrated the ability to perform and interpret a large variety of ultrasound exams, and a growing body of literature supports the use of point-of-care ultrasound in developing nations. We review, by region, the existing literature in support of ultrasound use in the developing world and training guidelines currently in use, and highlight indications for emergency ultrasound in the developing world. We suggest future directions for bedside ultrasound use and research to improve diagnostic capacity and patient care in the most remote areas of the globe.

## Background

It is generally accepted that in rural and remote areas of low- and middle-income countries (LMICs) diagnostic imaging is often insufficient, and in some instances completely lacking [1]. Over the past decade, however, the use of clinician-performed, hand-carried, bedside ultrasound has gained increasing popularity as a useful imaging modality worldwide, helping to boost the diagnostic capacity of rural district hospitals in resource-limited settings. The increase in ultrasound services provided by nonradiologists is likely due to several factors, including the increased affordability, availability, portability and durability of ultrasound machines. In addition, machine design has become more user-friendly for novice users with fewer knobs and streamlined design for quick comprehension of key features. Many new laptop-based machines are now in production. Improvements in battery life for hand-carried machines, and the lack of film, chemical developers and dedicated technicians, allow for use of ultrasound in health missions to remote areas of the developing world. Because of this evolution in technology and the growing body of literature to support its use, ultrasound has gained increasing recognition as a valuable diagnostic tool for resource-limited settings by the ministries of health in LMICs, several non-governmental organizations and the World Health Organization (WHO).

### Experience and prior study

There have been a multitude of small studies depicting novel uses of ultrasound in the developing world, but only a few studies have looked at the impact of ultrasound use on clinical management and patient outcomes, and whether ultrasound may be a sustainable modality for use in LMICs.

In a study from Rwanda, ultrasound was introduced at two rural district hospitals, and the impact on patient care was assessed by asking providers to identify if ultrasound changed patient management plans. Of the first 345 ultrasounds performed, the majority of scans were performed for obstetrical purposes (102), followed by abdominal (94), cardiac (49), renal (40) and pulmonary (36), along with a few procedural usages, soft tissue and vascular exams. In 43% of patient cases, ultrasound findings changed the initial patient management plan, with the most common changes cited as: performing a surgical procedure, medication changes, clinic referral and canceling of a planned surgical procedure [2].

In another study by Kotlyar et al., ultrasound changed patient management in 62% of cases at the major tertiary care center in Monrovia, Liberia. The greatest impact on patient management was seen with first trimester obstetric ultrasound, followed by FAST, cardiac and second/third trimester ultrasound exams; the smallest impact was seen in RUQ and gynecologic studies [3].

In another study of patients in the Amazon jungle, a group of American emergency physicians found that ultrasound examinations changed treatment in 28% of patients, including appropriate referrals for more definitive care in some cases and avoiding a potentially dangerous 2-day evacuation for additional medical care in others [4]. The consulting physician's differential diagnosis was narrowed after reviewing the ultrasound results in 72% of cases, with diagnostic certainty achieved in 68% of cases.

In 2004, a study by radiologists sought to demonstrate that portable ultrasound could enhance the medical management and clinical outcomes of patient care in a variety of clinical settings (surgeon's office, hospital operating room and clinics) in the Sekondi-Takoradi area, Ghana. In clinic settings, the most frequent ultrasound examinations were musculoskeletal (46%), with the remainder being obstetric, pelvic and genitourinary. In the hospital setting, abdominal, pelvic and genitourinary ultrasounds were the most frequent exams done to assess bladder masses, prostate and uterine size, and kidney abnormalities. A total of 67 ultrasound examinations were performed with abnormal findings in 54 (81%). One hundred percent of these abnormal ultrasounds were thought to add to the clinical diagnosis, and 40% (27) influenced the outcome or decision regarding treatment for these patients [5].

Similar results have also been seen in larger studies. In western Cameroon, the Ad Lucem Hospital of Banka-Bafang conducted a retrospective review of 1,119 ultrasound examinations and their effect on diagnosis and treatment. Abnormal findings were present in 78% of the cases, and 67.8% of the ultrasounds were judged to be useful for diagnosis, while only 4% were felt to be noncontributory. Ultrasound provided the diagnosis in 31.6% of the cases, and confirmed a prior diagnosis or allowed a differential diagnosis to be excluded in 36.2% of the cases. In a subpopulation of confirmed diagnosis (via tissue pathology, additional imaging tests, endoscopy, surgical specimen or laboratory diagnosis), approximately half of the diagnoses made by ultrasound had not been previously considered [6].

Despite its limitations, the impact of ultrasound is beginning to become clear, and this tool has become indispensable for the examination of cardiac, abdominal, obstetric, vascular, traumatic and musculoskeletal complaints in the developing world. Given the prevalence of poverty-related diseases, such as tuberculosis, malaria and dehydration due to diarrheal illness, in the developing world, it is no surprise that emerging uses for ultrasound in LMICs include these diseases [7-9]. We summarize here, by region, some of the highlights of diagnostic ultrasound research for specific diseases in resource-limited settings in LMICs.

### Africa

#### Egypt

Intrauterine growth retardation (IUGR) is a major contributing factor to perinatal mortality and morbidity in developing countries, and ultrasound may play an important role in early identification of pregnant mothers at risk. In 1988, Mahran et al. demonstrated an 11.8% rate of neonatal growth retardation in 828 pregnant women in Cairo, Egypt. In this group, antenatal ultrasound was able to predict 89.7% of these cases, while only 34.7% were predicted by fundal palpation [10].

#### Gambia

In a 2004 study in Gambia, physicians used a hand-held ultrasound to identify high-risk patients with cardiovascular disease and hypertension. Of the 1,997 patients seen, 17% (342) were found to have elevated blood pressure, and all of these patients underwent echocardiography to identify left ventricular hypertrophy, as a marker for those at highest risk of a cardiovascular event. Sixty-five percent of this hypertensive population demonstrated left ventricular hypertrophy by ultrasound and were started on antihypertensive medications. Patients with borderline hypertension also underwent a cardiac ultrasound examination and were started on antihypertensive medications only if they had evidence of left ventricular hypertrophy. Through this screening and the identification of high-risk hypertensive patients, ultrasound enabled a more effective use of limited healthcare resources [11].

#### Tanzania

In a district hospital in Karagwe, Tanzania, ultrasound services were studied to determine the impact on obstetric care. Nurse midwives, trained in basic obstetric ultrasound, were available to perform studies 24 h/day, whereas specialized ultrasonographers performing advanced ultrasound (including fetal biometrics) were only available during daytime hours. Five hundred forty-two patients with suspected abnormal findings were scanned over 1 year. When evaluating for twins, fetal heart rate or fetal positioning, the basic exam performed by the midwives had 100% agreement with the sonographer. Overall, ultrasound aided in the diagnosis of 39% (212) of patients and changed management plans in 22% (121). This study demonstrated that 24-h availability of basic obstetric ultrasonography performed by midlevel providers could be implemented in a rural hospital setting to lessen the workload of a specialist sonographer while improving patient care [12].

#### Zambia

In rural Zambia, 21 midwives participated in a pilot program for focused obstetric ultrasound to determine whether ultrasound skills could be imparted to nurse midwives. Obstetric ultrasound instruction given by ultrasound fellowship-trained emergency physicians included fetal presentation, fetal heart rate, placental location, number of gestations and assessment of gestational age. Over the 6-month training period, 441 ultrasounds were performed, with the main abnormal findings being non-vertex presentation (61%), multiple gestations (24%) and no fetal heart rate (8%). Ultrasound findings prompted a change in the clinical decision-making in 17% of cases. At the 1-year follow-up, ultrasound use continued, with an average number of ten ultrasound examinations per week per midwife, and 100% of the midwives reported that ultrasound helped their practice and changed their management [13].

#### Zambia/Congo

In a large retrospective study from the 1990s, abdominal ultrasound was used to define ultrasound findings of HIV through evaluation of 900 HIV-positive adults in the major tertiary referral hospitals of Lubumbashi, Congo, and Lusaka, Zambia. Ultrasound exams were performed by local practitioners for evaluation of various complaints, including pain, fever and organomegaly, and results of these ultrasounds were compared to age and sex-matched HIV-negative patients. Compared to HIV-negative patients, those with AIDS who underwent an ultrasound examination had significantly higher rates of splenomegaly (24% vs. 35%), hepatomegaly (22% vs. 35%), retroperitoneal and mesenteric lymphadenopathy (11% vs. 31%), biliary tract abnormalities, such as gallbladder wall thickening (12% vs. 25%), gut wall thickening (5% vs. 15%) and ascites (9% vs. 22%). The authors concluded that focused abdominal ultrasound in patients with HIV and AIDS can be a useful tool for diagnosing associated complex gastrointestinal pathology [14].

#### Malawi

Uncertainties regarding accurate gestational age may contribute to the difficulty in accurately assessing the role preterm birth plays in neonatal mortality in the developing world. Ultrasound may help to characterize the true magnitude of this public health concern. In a 2005 study, local practitioners performed ultrasound exams on 512 pregnant women prior to 24-week gestation presenting for prenatal care at a rural health center (Namitambo) or hospital (Thyolo) in Malawi, and provided an estimation of their gestational age. In this cohort, 20.3% of mothers delivered prematurely prior to 37 weeks of gestation, and these infants born between 32 and 37 weeks were twice as likely to die as their full-term counterparts (6.9% vs. 3.4%). This study introduces the idea that early obstetric ultrasound may allow for a more accurate assessment of the actual gestational age at the time of birth, thus demonstrating the true prevalence of preterm birth in the developing world [15].

#### South Africa

South African hospitals experience some of the highest trauma volumes in the world, and most have limited imaging capabilities, leading to significant diagnostic and therapeutic challenges. A study at the Ngwelezane Hospital, a busy referral center in rural KwaZulu-Natal, South Africa, examined the use of the FAST (focused assessment with sonography in trauma) exam on blunt and penetrating trauma victims. Over a 12-month period, 72 FAST scans were performed (52 for blunt trauma, 20 for penetrating trauma) with 15 positive scans (20.8%). The overall specificity of the FAST scan was 100%, with a sensitivity of 71.4%, but its sensitivity in penetrating trauma alone was much poorer at 62.5%. This study highlights the valuable role FAST scanning can play in the rapid assessment and timely transfer of appropriate trauma patients to referral hospitals [16].

### Asia: India

The diagnosis of abdominal tuberculosis is often difficult in the developing world due it its vague clinical features, mimicry of other diseases, and expensive/time consuming workup with CT scan and laparotomy. In Uttar Pradesh, India, investigators sought to assess the accuracy of ultrasound for diagnosis of abdominal tuberculosis in symptomatic patients co-infected with HIV. A retrospective review of 2,543 patients evaluated ultrasound use in an antiretroviral clinic. Patients with persistent fever, change in bowel movements, diarrhea or abdominal distention received an ultrasound evaluating Tb-related pathology such as lymphadenopathy (nodes >15 mm), organomegaly or multiple small abscesses/hypoechoic lesions in abdominal solid organs, bowel wall thickening, peritoneal nodules, mesenteric thickening or ascites as well as other abdominal pathologies. Of the 2,453 patients in an antiretroviral clinic, 373 were evaluated by ultrasound, of which 244 showed features suggestive of abdominal tuberculosis, with lymphadenopathy as the most common finding (64.8%), followed by splenomegaly (27.9%), hepatomegaly (20.1%) and bowel wall thickening (6.1%). Ultrasound was repeated at the end of antitubercular therapy, showing resolution of abnormal findings, which suggests this imaging modality may be useful in the diagnosing and monitoring of HIV-positive patients with abdominal tuberculosis [9].

### North America: Mexico

In an attempt to characterize cardiac disease prevalence in the developing world, American cardiologists used hand-carried ultrasound in outpatient clinics in rural San Blas and El Fuerte, Mexico. They performed cardiac ultrasound exams on 126 patients referred to the clinic by their primary care doctors for hypertension, chest pain, dyspnea, edema, murmurs, suspected congenital abnormalities, palpitations and syncope, of which 68% (86) exams were abnormal. The most common abnormal findings included significant valvular disease, left ventricular hypertrophy, dilated aortic root/increased chamber size, congenital abnormalities (including bicuspid aortic valve, PDA, atrial and ventricular septal defects), ventricular systolic dysfunction and regional wall motion abnormalities. In 93% of patients, hand-held ultrasound provided useful information that helped to clarify the clinical problem, in 63% of cases, ultrasound confirmed the cardiac origin of a symptom and in 90% of cases it made conventional echocardiography unnecessary. Hand-held ultrasound can provide useful diagnostic information in the evaluation of patients with potential cardiac disease in resource-limited settings [17].

### Training in ultrasound

As demonstrated in the studies highlighted above, ultrasound can significantly impact the diagnosis and management of patients in LMICs. However, ensuring the sustainability of ultrasound programs in resource-limited settings will also require the implementation of successful training programs for local practitioners and the development of markers for quality assurance.

In 1998, the WHO established standards in ultrasound training and recommended that an appropriate curriculum be adopted for the training of practitioners in the use of diagnostic ultrasound [18]. However, there have been no standardized approaches to length of training, curriculum for general practitioners, qualifications of trainers or mechanism of training published in the literature.

For example, successful ultrasound training courses implemented in LMICs have varied in length from as little as 4 days to several months. In 2005, Adler et al. introduced a portable ultrasound machine into the Lugufu refugee camp in Kigoma District, Tanzania, and conducted an intensive 4-day ultrasound training course for the local healthcare providers [19]. Shah et al. conducted a training course in Rwanda with a length of 9 weeks in 2008 [20].

Trainees in the various study populations have ranged from clinical officers, nurses and nurse midwives to fully trained physicians. Trainers have similarly varied in these studies, including resident physicians, emergency physicians, radiologists, cardiologists and ultrasound-fellowship trained emergency physicians.

The curriculum and method of training described in the literature for general ultrasound in LMICs range from broad to focused, depending on the goals of the program and research study. In the Adler study [19], the training course consisted of morning interactive classroom sessions addressing basic ultrasound physics, use of ultrasound machine knobs and reviews of specific clinical ultrasound applications, including FAST, abdominal aorta, hepato-biliary, first trimester pregnancy ultrasound, fetal position and gestational age, ultrasound-guided procedures, soft tissue, basic cardiac exam and renal ultrasound. The afternoons and early evening were spent doing hands-on evaluations of inpatients and outpatients at the Lugufu hospital and clinics. During the 2-year study period, 547 ultrasound exams were performed on 460 patients. The most common ultrasound exam performed was for obstetrical purposes (24%), followed by abdominal ultrasounds (22.7%), pelvic ultrasound (21.9%), renal (9.9%) and RUQ exams (9.0%). Building upon this training model, Shah et al. [20] developed an ultrasound training program in rural Rwanda that spanned 9 weeks, and included lectures, hands on practice sessions and scan time during daily ward rounds conducted by the instructor with the local practitioners. Topics covered included ultrasound physics, obstetric ultrasound (first trimester ectopic and molar pregnancies, estimation of gestational age, and evaluation of the fetal position, cervix and placenta), echocardiography (rheumatic disease, mitral stenosis, estimation of ventricular function and pericardial effusion), hepatobiliary ultrasound (including evaluation for amebic abscess, echinococcal cysts, cholecystitis), renal ultrasound, and advanced topics such as deep venous thrombosis, vascular access, skin and soft tissue evaluation and procedural guidance. This study added bimonthly review sessions, and appointment of an "ultrasound coordinator" at each hospital site who was entrusted with the care of the ultrasound machine, gel and supply ordering, ultrasound logbook upkeep and uploading images from the ultrasound to their personal computer. Through this ultrasound coordinator, physicians were able to send images via e-mail for ongoing quality assurance after the training period ended.

Overall, the available literature suggests that a short but intensive training period is sufficient for preparing clinical officers, nurses and physicians alike to perform basic ultrasound exams, especially if the training program includes both lecture and practical experience, and provides opportunity for continued upkeep of skills through review sessions and ongoing quality assurance after the training period ends.

### Indications for ultrasound in resource-limited settings

While a long list of potential indications for ultrasound in low resource settings exists and has filled a textbook [21], we focus here on the main emergency ultrasound indications that may help reduce morbidity and mortality in the developing world.

### Emergency obstetric ultrasound

Ultrasound can be a valuable tool in all trimesters of pregnancy. In the first trimester of pregnancy, ectopic pregnancy is a leading cause of mortality in women in LMICs, requiring early identification and prompt intervention. Since clinical signs and symptoms are not reliable, ultrasound can play a pivotal role in its diagnosis. Early in their pregnancy, many women seek care for abdominal pain and/or vaginal bleeding. Bedside ultrasound by Emergency Physicians (EPs) in the United States has provided rapid exclusion of ectopic pregnancy by identifying intrauterine pregnancy (visualizing the yolk sac or fetal pole). In one study, instituting a protocol for bedside ultrasound by EPs in evaluating patients with first trimester bleeding decreased the delay to diagnosis of ectopic pregnancy from 43% to 29% and decreased the rate of missed ruptured ectopic pregnancy from 50% to 9% [22]. Another study, evaluating EPs' bedside ultrasound in 125 patients showed a 96% agreement with formal radiology department ultrasound, suggesting this is a rapidly learned skill for non-radiologists and non-obstetric specialists. EP ultrasound for diagnosing ectopic pregnancy had a sensitivity and specificity of 90% and 88%, respectively, and a negative predictive value of 100% [23]. Adding a right upper quadrant view to search for free fluid in Morison's pouch aids in the diagnosis of a ruptured ectopic pregnancy. Free fluid is a good predictor of necessitating operative intervention and decreases time to such inventions [24,25].

Estimating gestational age in second and third trimester pregnancy can be helpful in the diagnosis and management of preterm labor and can be performed successfully by non-obstetric specialists with a high degree of sensitivity. In one study, after a didactic session and a proctored exam, eight EPs evaluated a sample of pregnant patients (14-40 weeks) showing a high correlation of fetal biometrics obtained by EPs compared with the ultrasound technicians (correlation of 0.96 for biparietal diameter and 0.97 for femur length) [26]. In this study, ultrasound was more accurate than fundal height measurement in determining fetal viability (fetal age >24 weeks) with an accuracy of 96% versus 80%. Internationally, obstetric ultrasound has been used to train local healthcare workers and midwives. As previously discussed, focused maternal ultrasound training for midwives has been successful in Zambia [13], and other studies from Burma and Bangladesh show local health practitioners can be trained in estimating gestational age using ultrasound. In a refugee camp on the Thai-Burmese border, four local healthcare workers were trained in OB ultrasound in 2009. Evaluation of fetal biometrics for 349 patients showed good inter-observer reliability between healthcare workers and the expatriate doctor [27]. In Bangladesh, nine paramedics (with no previous ultrasound experience) were trained and evaluated on their accuracy of measuring fetal biometrics. Results of the evaluation of 180 pregnant women (7-31 weeks) showed an inter-observer error rate that was quoted to be within an acceptable range for ultrasound technicians [28].

In addition to evaluating ectopic pregnancy and estimating gestational age, bedside ultrasound can also be used to evaluate gynecological infections, i.e., tubo-ovarian abscess, by identifying complex adnexal masses, pyosalpinx or echogenic pelvic fluid on ultrasound [29]. These specific obstetric and gynecological ultrasound exams have a high yield for the diagnosis and management of reproductive-age females in the developing world.

### Trauma ultrasound

FAST (Focused Assessment with Sonography in Trauma) has been used routinely in the management of trauma patients worldwide as a rapid, noninvasive way to evaluate patients with thoracoabdominal trauma. The FAST scan can be performed within minutes at the bedside and can help in resource-limited settings with decisions regarding which patients require immediate operative care. The FAST exam has been shown to decrease the time to operative intervention in a randomized controlled trial as compared to a standard clinical evaluation. Patients that received FAST had a 64% decrease in the time to operative intervention, and decreased complication rates and hospital length of stay [30]. The sensitivity of FAST scans in detecting intraperitoneal hemorrhage is 75-78% and its specificity is 98-100%, suggesting it is a useful tool to confirm the presence of hemoperitoneum and hemopericardium [31,32]. Clinically, the FAST scan is particularly beneficial in the hypotensive trauma patient whose source of hypotension is unclear [33].

In addition to its use in resource-limited settings during routine clinical care, FAST is also useful in disasters and mass casualty scenarios. When evaluating multiple severely injured patients in a disaster setting, FAST can aid in rapid triage of injured patients and guide operative care. For example, FAST during wartime in Lebanon was employed as a tool for soldiers suspected of having abdominal injuries to help triage them to operative intervention, computed tomography (CT) or clinical observation [34]. FAST has also been used by medical relief workers during multiple natural disasters in the past 2 decades, including the earthquake in Armenia in 1988 [35], Wenchuan, China, in 2008 [36], and Haiti in 2010 [37], and the tsunami in Indonesia in 2004 [38]. FAST is also valuable in a resource-limited setting where there is limited access to computed tomography. The utility of FAST was evaluated in a government hospital in rural KwaZulu Natal in South Africa, as mentioned above [16].

Ultrasound evaluation for pneumothorax has been added to the FAST exam (Extended FAST) and has proven to be a powerful additional adjunct to trauma ultrasound. Studies comparing ultrasound with supine chest x-ray has demonstrated that ultrasound has greater sensitivity in the diagnosis of pneumothorax. The sensitivity and specificity of ultrasound range between 86.2-98.1 and 97.2-100%, respectively, while the sensitivity and specificity of chest x-ray are between 27.6-75.5% and 100%, respectively [39,40]. A study from the military literature evaluating the performance of non-physicians (physician assistants, medics, veterinary technicians and a food service inspector) performing ultrasound for pneumothorax showed great success. After a brief instructional session, 22 non-physicians evaluated 44 hemi-thoraces of porcine models (some with induced pneumothoraces), showing a sensitivity of 95.4%, specificity of 100%, PPV 100% and NPV 95.6% [41]. Evaluation of pneumothorax has been extended to prehospital care in Europe [42], suggesting that ultrasound for pneumothorax is an easily acquired skill, even for novice sonographers.

Despite the encouraging literature, it is important to remember that all ultrasound, including the FAST exam, is dependent on the training and experience of the clinician. A study evaluating non-radiologist physicians (surgeons and emergency medicine physicians) found that while the initial error rate for FAST scans was 17%, it fell to 5% after performing ten exams [43]. Another study evaluating surgeons, radiologists and technicians found that the learning curve for FAST leveled off after 30 exams [44].

### Cardiac ultrasound

Focused echocardiography has an important role in assessing patients with cardiovascular compromise. It is useful in diagnosing pericardial effusion, assessing left ventricular ejection fraction, assessing volume status in patients with shock and delineating the etiology of cardiovascular collapse. In one study in the US evaluating patients with dyspnea of unclear etiology (i.e., after ruling out congestive heart failure, pneumonia, COPD, pulmonary embolism), pericardial effusion was found in 13.6% of patients [45]. The incidence of such effusions may be higher in communities where HIV and tuberculosis are more prevalent. Research supports the ability of non-cardiologist physicians to accurately diagnose pericardial effusions. In a large study evaluating 515 high-risk patients with dyspnea, 103 had a pericardial effusion. EPs' bedside echocardiography obtained the diagnosis with a sensitivity and specificity of 96% and 98%, respectively [46]. In a hemodynamically unstable patient, early diagnosis of cardiac tamponade can expedite bedside ultrasound-guided pericardiocentesis [47,48].

Ultrasound is also used in the evaluation of shock as a measure of cardiac contractility. The literature has shown that EPs' estimation of the left ventricular ejection fraction correlates well with cardiologists' estimation of LVEF [49,50]. In patients with pending cardiovascular collapse or cardiac arrest, ultrasound is able to identify reversible or correctable causes. In a study of 20 patients in PEA or near PEA arrest, bedside echocardiography diagnosed 8 patients with pericardial effusion, 3 of which were in tamponade requiring emergency pericardiocentesis [51]. In cardiac arrest, ultrasound can guide when to cease resuscitation efforts because of the high correlation between lack of organized cardiac motion on ultrasound and lack of return of spontaneous circulation [52,53].

### Ultrasound evaluation for deep venous thrombosis and pulmonary embolism

Evaluating for deep vein thrombosis (DVT) is useful in patients with leg swelling/pain, crush injuries, prolonged immobilization, recent surgery and other pertinent risk factors. In resource-limited settings, due to the hypercoagulability of HIV-positive patients and the lack of routine prophylactic anticoagulation of hospitalized patients, bedside diagnosis of DVT is especially important in providing timely care. Ultrasound evaluation for DVT has been shown to be successful by nonvascular specialists ranging from novice to advanced users of ultrasound, and can be performed in just a few minutes at the patient's bedside [54,55]. A study evaluating 56 emergency clinicians (attending physicians, residents and midlevel providers) after didactic training showed an initial sensitivity and specificity of 70% and 89%, respectively. The sensitivity improved to 100% for clinicians who performed three or more scans [56]. A more recent study showed that physicians trained with a brief focused module could achieve sensitivity of 100% and specificity of 99% for detection of DVT with bedside ultrasound [57], with similar studies supporting a high correlation with radiologically performed DVT evaluations. In addition, although echocardiography cannot effectively rule out pulmonary embolism, in the case of a massive or sub-massive pulmonary embolism, echocardiography may show right ventricular enlargement, tricuspid regurgitation, and paradoxical septal shift into the left ventricle or a ventricular thrombus [58,59].

### Ultrasound in surgical emergencies

In settings with limited access to surgical care and computed tomography, ultrasound can help to identify true surgical emergencies, allowing efficient use of resources. Given the burdens of patient transportation in the developing world and cost associated with unnecessary consultations and imaging tests, bedside ultrasound can help streamline diagnosis and patient care.

While access to specialized vascular surgery may be limited, ultrasound diagnosis of abdominal aortic aneurysm (AAA) as a cause for abdominal pain may allow for rapid identification of surgical candidates and save resources for further diagnostic workup once a diagnosis has been reached. Testing for syphilis, a cause of AAA, can be initiated for the patient and their partners, and in cases where vascular surgery is not available, other risk factors such as hypertension can be controlled to reduce the risk of rupture. Prior studies of ultrasound for diagnosis of AAA have shown high rates of sensitivity and specificity [60,61].

Surgical hepatobiliary diseases such as cholecystitis, may be rapidly diagnosed by non-radiologist healthcare providers using ultrasound. The emergency medicine literature suggests clinicians performing bedside ultrasound can attain a high sensitivity and specificity for detection of cholecystitis compared with formal ultrasound radiology [62,63]. Ultrasound can also be used to diagnose liver abscesses with a sensitivity of 86%, to guide aspiration/percutaneous drainage and to follow resolution during treatment [64,65].

While evaluation of appendicitis in the developed world relies heavily on computed tomography and surgical consultation, in resource-limited settings the diagnosis is often made by generalist clinicians based on history and physical exam alone. Ultrasound may be useful in these settings to improve diagnostic capacity, especially in pediatric patients or patients in whom a non-therapeutic appendectomy would be more risky. In a meta-analysis comparing ultrasound and CT (pooling 9,356 children and 4,341 adults), ultrasound demonstrated a sensitivity and specificity of 88% and 94% in children and 83% and 93%, respectively, in adults [66]. It is important to note that the diagnosis of appendicitis cannot be excluded unless a normal appendix is visualized.

### Procedural ultrasound

Obtaining peripheral venous access can be a challenge in any setting in patients with obesity, vasculopathy, hypovolemia/dehydration or history of intravenous drug use. In patients where the traditional approach has failed, ultrasound can be used to visualize a target vein. Physicians have demonstrated a success rate of 91- 97% with a decreased time to cannulation and improved patient satisfaction, and a very low risk of accidental arterial puncture [67,68]. Nurses and technicians trained in this technique have demonstrated an 87% and 78.5% success rate, respectively [69,70].

Given the prevalence of tuberculosis-related pleural effusions and ascites in hospitalized patients in LMICs, ultrasound guidance for these procedures may improve safety and success. A study evaluating ultrasound-guided thoracentesis in 605 patients demonstrated a decreased risk of pneumothorax with a rate of 2.5% compared with previously quoted rates of 4-30% [71]. A study evaluating ultrasound-guided paracentesis demonstrated success in 95% of patients who had ultrasound compared with 61% of patients who did not receive ultrasound. Ultrasound was able to identify if patients had inadequate fluid for the procedure and provided an alternative diagnosis in two patients with abdominal swelling: a large cystic mass in one and ventral hernia in another [72].

A safe alternative to conscious sedation in settings where airway and anesthesia support may be limited is the use of ultrasound guidance for regional anesthesia. Ultrasound-guided nerve blocks can be used to provide regional anesthesia in complex laceration repairs, orthopedic fracture/dislocation reductions and operative procedures. Ultrasound-guided nerve blocks improve success rates and reduce the number of needle passes, dosage of local anesthesia and complication rates [73].

### Future directions

While prior research has clearly shown that the introduction of bedside ultrasound can have a significant impact on clinical management in resource-limited settings, and that providers in these settings can be rapidly trained to use ultrasound for basic diagnostic and procedural indications, many questions still remain for future investigation. In particular, various training models for emergency ultrasound in resource-limited settings should be directly compared through controlled studies to determine the most effective and least time- and cost-intensive methods for training general providers in these settings. In addition, more research is needed to determine whether remote review of ultrasound images can aid in the upkeep of ultrasound skills by local practitioners while also providing quality assurance for images obtained in resource-limited settings. In order to create greater uniformity in the quality of bedside ultrasound performed in resource-limited settings, professional organizations and inter-governmental organizations should work together to create standard curricula and international mechanisms for certification in basic ultrasound for clinicians.

Furthermore, additional research is needed to help clarify the utility of diagnostic ultrasound for a host of different diseases and conditions. While most prior research has focused on determining the accuracy and reliability of ultrasound in resource-limited settings, future research should focus on the effectiveness and cost-effectiveness of ultrasound in these settings (i.e., does the introduction of ultrasound reduce morbidity and mortality while also helping reduce overall costs). Finally, there are several areas where ultrasound may prove useful for particular indications in LMICs that have not been well studied in high-income countries, either because the disease process is not prevalent or better diagnostic tests are already available. Below is a list of several promising new indications for diagnostic ultrasound in LMICs.

### Hemorrhage

Hemorrhage is the leading cause of maternal mortality in low- and middle-income countries worldwide [74]. Access to blood transfusion is considered a key component of Comprehensive Emergency Obstetric Care (EmOC) and should be available at the heath center or district hospital level for all women suffering from hemorrhage during pregnancy [75]. In addition to obstetric emergencies, blood loss remains an important complication of trauma in the resource-limited settings. Injuries are among the top ten leading causes of death and disability in the developing world, and rates of death from nearly all types of injuries are higher in developing countries than in developed countries [76,77]. However, in resource-limited settings, the availability of blood for transfusion is likely to be limited, and the blood that is available may not be comprehensively screened for infectious diseases such as HIV or hepatitis B. Therefore, it becomes incredibly important to have an accurate estimate of blood loss in both trauma patients and obstetric patients in order to ensure that those who truly need lifesaving blood transfusions get them rapidly, but precious and potentially dangerous blood transfusions are only used when absolutely necessary.

However, blood loss in both obstetric and trauma patients may be difficult to assess clinically. Patients may not develop supine or even postural tachycardia or hypotension until they have lost a significant amount of blood volume [78]. In addition, laboratory testing of hemoglobin or other measures of blood loss such as base excess may not be available in some resource-limited settings; this is especially true during disasters and humanitarian emergencies. In these settings, ultrasound of the IVC may prove to be a useful adjunct for assessing blood loss. Lyons et al. measured IVC expiratory diameter in healthy adult volunteers before and after donating 450 cc of blood. The average IVC diameter was 17.4 mm pre-donation and 11.9 after donation, which was significantly different between groups [79]. In a study of 88 trauma patients presenting to a single trauma center, both IVC diameter and percent IVC collapse with respiration (Caval Index) correlated strongly with the presence of shock [80]. In another study, Yanagawa et al. found that in trauma patients presenting with shock who initially responded to resuscitation, those with a smaller IVC diameter (average 6 mm) were more likely to drop their blood pressures again compared to those with a larger IVC (average 11 mm); this was a better predictor than heart rate in these patients [81]. In both trauma and obstetric patients, multiple ultrasound measurements of IVC may be especially useful in order to get a sense of blood loss over time.

### Sepsis

Perhaps the most important initial treatment for septic patients is to provide adequate resuscitation with intravenous fluids (normal saline or lactated Ringer's), and the International Guidelines for Management of Severe Sepsis and Septic Shock recommend initial resuscitation to a CVP between 8 and 12 mmHg for all patients with sepsis [82]. While CVP can be measured directly by placing a central venous catheter with its tip in the right atrium, providers in resource-limited settings are unlikely to have access to this technology. Several studies have found that both the IVC size and Caval Index (CI) correlate well with CVP. In a study of septic patients in the Intensive Care Unit (ICU), Kircher et al. found that a CI of <50% had the best sensitivity (87%) and specificity (82%) for detecting a CVP > 10 [83]. In a separate population of ICU patients with sepsis, Feisel et al. found that a CI of >12% correctly identified most patients who would be "fluid responders," meaning that their cardiac output would increase in response to a bolus of fluid. Those with CI <12% had no increase in cardiac output with additional fluid, with a positive and negative predictive value of 93% and 92%, respectively [84]. In a study of emergency department patients with sepsis, Nagdev et al. found that a CI greater than 50% had a sensitivity of 91% and specificity of 94% for detecting a CVP <8 mm Hg, with a positive likelihood ratio of 15.5 and a negative likelihood ratio of 0.1 [85].

### Dehydration

Dehydration due to diarrhea is one of the leading causes of death in children under 5 in the developing world, responsible for nearly 2 million deaths (19% of all child deaths) each year [86]. In addition, epidemic diarrhea can kill thousands of children and adults in a matter of days or weeks in the setting of disasters and humanitarian emergencies, as evidenced by the recent cholera epidemic in Haiti. As the severity of diarrhea can vary widely in these settings, accurately assessing dehydration status remains a crucial step in preventing mortality from this disease. While patients with severe dehydration require immediate treatment with intravenous fluids to prevent hemodynamic compromise, organ ischemia and death, a large meta-analysis found that children with mild to moderate dehydration have a significant reduction in hospital length of stay and fewer adverse events when treated with relatively inexpensive oral rehydration solution (ORS) as compared to treatment with costly intravenous fluids [87]. Unfortunately, the diagnostic tools available to physicians and other providers to assess the degree of dehydration in children with diarrhea are limited. A recent meta-analysis of 13 studies found that no individual clinical sign or symptom demonstrated adequate sensitivity, specificity or reliability for detecting dehydration in children [88]. A similar meta-analysis in adults found clinical measures of dehydration to be even less useful [78].

Ultrasound may be a better measure of dehydration in patients with acute diarrhea. In children, since the size of a the IVC varies with age, research is currently focusing on the aorta to IVC ratio as a measure of volume status; the aorta provides an internal control for IVC since it does not change much in size with dehydration. In a recently published study of 52 children presenting to hospitals in rural Rwanda with diarrhea and/or vomiting, the aorta/IVC ratio had an area under the ROC curve statistically different from the reference line [area under the curve (AUC) = 0.76; 95% CI = 0.62 to 0.90]. Using the best cutoff of 1.22, aorta/IVC ratio had a sensitivity of 93% (95% CI = 81% to 100%), specificity of 59% (95% CI = 44% to 75%), LR+ of 2.3 (95% CI = 1.5 to 3.5) and LR- of 0.11 (95% CI = 0.02 to 0.76) for detecting severe dehydration [7]. In a similar study of 71 children presenting to an urban academic emergency department in the United States, the IVC/aorta ratio was found to have an AUC of 0.73 (95% CI = 0.61 to 0.84). An IVC/aorta cutoff of 0.8 produced a sensitivity of 86% and a specificity of 56% for the diagnosis of significant dehydration [89].

### Edema in children

Edema is a common presenting complaint among children in low-income countries. It can be due to hypervolemia, such as in the settings of congestive heart failure secondary to rheumatic heart disease or renal insufficiency, or it can be due to low oncotic pressure from a nephrotic syndrome or protein-energy malnutrition (kwashiorkor). Indeed, patients with severe protein-energy malnutrition may appear edematous at the same time as they are actually intravascularly dehydrated, making it difficult to adequately assess their volume status on physical exam. Two studies from pediatric nephrology clinics in Poland and Israel suggest that ultrasound of the IVC may be useful for differentiating hypervolemia from edema secondary to low oncotic pressure, which could prove useful in guiding further diagnostic testing and management of patients in resource-limited settings.

### Cerebral malaria

Novel research from the pediatric acute care unit of Mulago Hospital in Kampala, Uganda, shows ultrasound of the optic nerve sheath diameter (ONSD) may be predictive of cerebral malaria in children. In this study, 33 children diagnosed with *P. falciparum *underwent targeted ultrasound examinations of optic nerve sheath diameter, color transcranial Doppler insonation of the cerebral vasculature, cardiac ultrasound and abdominal ultrasound to evaluate spleen size. Increased optic nerve sheath diameter was observed in one third of all patients with malaria and in 100% of the patients with cerebral malaria, and after successful treatment showed return to normal size [8].

## Conclusions

Clinician-performed bedside ultrasonography is emerging as a useful diagnostic tool for healthcare providers in resource-limited settings. Much of the research to date supports the use of this technology to guide management plans and procedures; however, comprehensive, long-term studies have not been performed. As further research emerges to evaluate the impact of ultrasound in resource-limited settings it will be possible to draw conclusions about the long-term sustainability of ultrasound programs in the developing world, target populations who may benefit most from ultrasound services, and further expand the indications for its use.

## Author details

Department of Emergency Medicine, University of Washington Medical Center,

Harborview Medical Center, 325 Ninth Avenue, Seattle WA, 98104, USA.

## Competing interests

The authors declare that they have no competing interests.

## Authors' contributions

All authors have made substantive contributions to this review article as they were all involved in the literature search, evaluation of the literature, writing and editing process. All authors read and approved the final manuscript.
